# Rapid Visualisation of Microarray Copy Number Data for the Detection of Structural Variations Linked to a Disease Phenotype

**DOI:** 10.1371/journal.pone.0043466

**Published:** 2012-08-17

**Authors:** Ian M. Carr, Christine P. Diggle, Kamron Khan, Chris Inglehearn, Martin McKibbin, David T. Bonthron, Alexander F. Markham, Rashida Anwar, Angus Dobbie, Sergio D.J. Pena, Manir Ali

**Affiliations:** 1 School of Medicine, University of Leeds, Leeds, United Kingdom; 2 Ophthalmology Department, St James's University Hospital, Leeds, United Kingdom; 3 Department of Clinical Genetics, St James's University Hospital, Leeds, United Kingdom; 4 Department of Biochemistry and Immunology, Universidade Federal de Minas Gerais and GENE - Nucleo de Genetica Medica, Belo Horizonte, Minas Gerais, Brazil; Karolinska Institutet, Sweden

## Abstract

Whilst the majority of inherited diseases have been found to be caused by single base substitutions, small insertions or deletions (<1Kb), a significant proportion of genetic variability is due to copy number variation (CNV). The possible role of CNV in monogenic and complex diseases has recently attracted considerable interest. However, until the development of whole genome, oligonucleotide micro-arrays, designed specifically to detect the presence of copy number variation, it was not easy to screen an individual for the presence of unknown deletions or duplications with sizes below the level of sensitivity of optical microscopy (3–5 Mb). Now that currently available oligonucleotide micro-arrays have in excess of a million probes, the problem of copy number analysis has moved from one of data production to that of data analysis. We have developed CNViewer, to identify copy number variation that co-segregates with a disease phenotype in small nuclear families, from genome-wide oligonucleotide micro-array data. This freely available program should constitute a useful addition to the diagnostic armamentarium of clinical geneticists.

## Introduction

With the development of high throughput technologies, genomics is rapidly moving into the clinical arena [1]. However, clinical genomics poses significant challenges to physicians, who need to be computer-literate if, as aptly expressed by Ware et al [2], they wish to “surf the wave of genomic opportunity”. Traditionally, bioinformaticians have used software that makes extensive use of command lines and LINUX operational systems. Clinicians, in contrast, need user-friendly graphical software that is preferably Windows-based and free. Consequently, we have tried to address these concerns with the development of CNViewer, a simple computer program for the visualization and analysis of human genomic copy number variations (CNVs).

Recently, structural genomic rearrangements have been found to be a major source of phenotypic variation [3], [4]. They may modify a gene's activity and expression by changing its copy number, altering its chromatin structure or by directly disrupting the structure of the transcriptional unit. Consequently, they may be a significant cause of genetic disease.

Although many chromosomal aberrations can be readily identified through karyotypic studies, conventional cytogenetic analysis cannot reliably detect rearrangements of genomic segments smaller than 3–5 million base pairs (Mb) [5]. For chromosomal rearrangements smaller than that, a number of techniques including fluorescent in situ hybridization [6], multiplex ligation-dependent probe amplification [7], array-comparative genomic hybridization (aCGH) [8] and microarray oligonucleotide hybridisation [9] have been developed. The first two depend on previous knowledge of the region to be scrutinized, which is only possible when a specific clinical suspicion exists. On the other hand, chromosomal micro-rearrangements vary in size and are often associated with non-specific phenotypes. Thus, there is a need for procedures that can screen the whole genome for subtle structural alterations and the only ones that meet this requirement are aCGH and oligonucleotide microarray hybridisation.

With the development of microarrays containing CNV probes, such as Affymetrix's SNP 6.0 genotyping microarray, it is possible to simultaneously genotype approximately 0.9 million SNPs and screen for copy number variation with approximately 1.9 million probes. Consequently, with the dual ability to genotype both SNPs and CNVs, oligonucleotide microarray analysis has been used extensively in genome-wide association studies (GWAS) [10], [11], [12].

To assist with the analysis of SNP and CNV microarray data, a number of algorithms have been implemented in various software applications [13], [14], [15]. Although, the error rate of microarray-derived CNV detection is believed to be greater than that observed with aCGH data [16], [17], the technique is becoming increasingly popular. The Canary (copy number genotyping), Birdseed (SNP genotyping) and Birdseye (CNV discovery) suite of algorithms implemented in the Affymetrix Genotyping Console [18], [19] perform genotyping and CNV detection in a multi-step manner.

**Figure 1:**
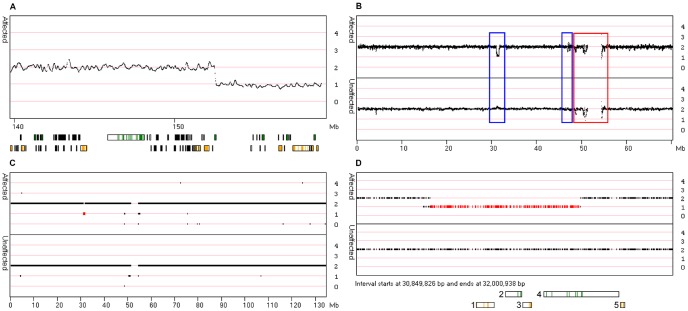
Detection of CNV using CNViewer. The ‘Smooth signal’ data for the tip of the long arm of chromosome 7 of **Patient One** has an extended run of values tending around 1, representing the presence of a 6.5 Mb deletion (Figure 1A). The position of genes in the interval is shown by the black rectangles below the main graph. The coloured blocks represent the location of the exons on the forward (green) and reverse (orange) strands. The ‘Smooth signal’ data, from a 70 Mb region of chromosome 11, for each individual in **Pedigree One** is overlaid on the upper (affected individuals) and lower graphs (unaffected siblings) in Figure 1B. The red box highlights regions where the CNVs are present in both affected and unaffected individuals, while the blue boxes identify CNVs that are present in the affected but not unaffected individuals. When the ‘CN state’ data for chromosome 11 is viewed with the ‘Show linked’ option selected, a single region starting at 31.7 Mb is highlighted (red bar, Figure 1C). When this region is expanded it can be seen that all the affected individuals contain a deletion, which is absent from the unaffected individuals (Figure 1D). This region contains 5 genes which are: 1 *DCDC1*; 2, *DNAJC24*; 3, *IMMP1L*; 4, *ELP4;* and 5, *PAX6*.

Initially, the CNV probe intensities are compared against a map of common known, copy number polymorphisms, allowing these CNVs to be typed whilst also enabling the CNV probe intensities to be grouped into clusters of inferred copy number. These clusters are then employed to aid in the genotyping of SNPs whose allelic copy number is expected to be 2 (homozygous for the probe-specific allele), 1 (heterozygotes) or 0 (homozygous for the alternative allele). Finally, a hidden Markov model, which uses the probe intensity and copy number data gathered while genotyping the SNPs and the common CNVs identifies regions of either rare or *de novo* CNV [18], [19]. Using such algorithms, it has been possible to identify 56% of common CNVs that contain 2 probes and up to 94% of common CNVs that span 20 probes. With a typical marker density of one probe per 1,600 bp, this corresponds to CNVs of approximately 3.2 or 32 Kb in length, respectively. When used to identify CNVs bio-informatically inserted into biologically-derived data, the method detected 10%, 51% or 97.5% of the synthetic CNVs, which contained 2 (∼3.2 Kb), 5 (∼8 Kb) and 10 (∼16 Kb) probes, respectively.

**Figure 2:**
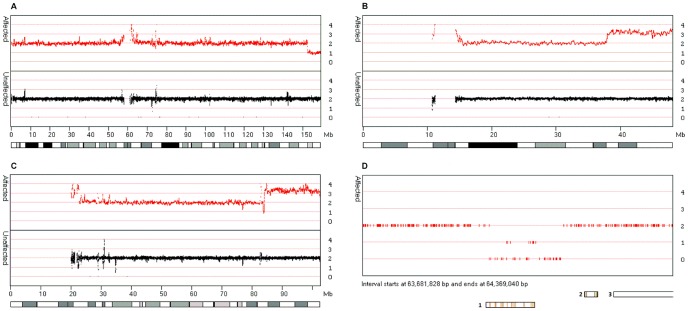
Identification of CNVs in patients 1, 2 and 3. Figures 2A to 2C show comparison of data from **Patients One** and **Two**, compared to data from 8 individuals unrelated to the patients. Figures 2A and 2B display ‘Smooth signal’ data for chromosomes 7 and 21, respectively, for **Patient One** and identify the location of a 7 Mb (152.1 to 159.0 Mb) deletion and a 10.2 Mb (36.8 to 47.0 MB) duplication. Figure 2C displays the ‘Smooth signal’ data for chromosome 15 of **Patient Two** and shows an 18.5 Mb (81.8 to 100.3 Mb) duplication. Figure 2D displays the ‘CN state’ data, from **Patient Three**, for an interval on chromosome 12 starting at 63.68 Mb and ending at 64.37 Mb**.** This clearly shows the presence of a 160 Kb homozygous deletion, which encompasses the *DPY10L2* gene locus (labelled 1), while not affecting the nearby *TMEM5* and *SRGAP*1 (labelled 2 and 3, respectively) genes.

While the CNV detection algorithm implemented by the Affymetrix Genotyping Console is able to detect the majority of CNV within an individual, the visualisation and identification of important CNVs may be quite difficult when using this or similar software applications. Consequently, we have developed CNViewer, a simple, free to use, user-friendly, Windows-based software tool for use by clinicians, which allows the rapid visualisation and detection of CNVs that may be linked to a disease phenotype. Also, when used with data from multiple members of a small pedigree, it can identify CNVs segregating with a disease phenotype.

**Figure 3:**
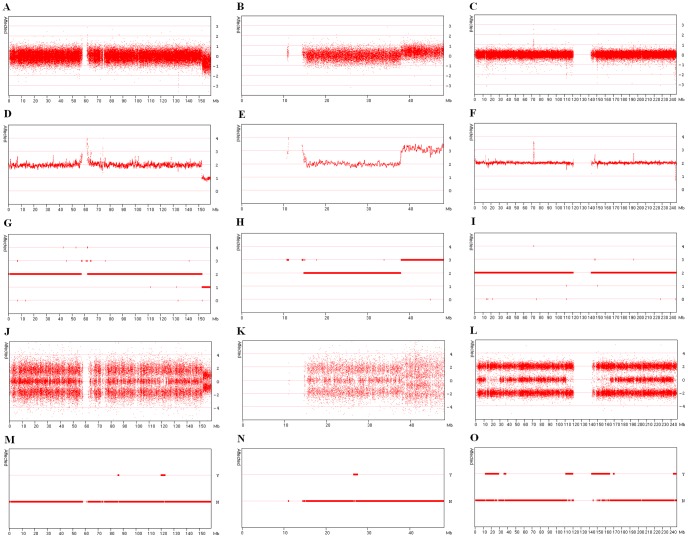
Visualisation of chromosomes containing a large deletion, duplication or autozygous segments. Figures 3A, 3D, 3G, 3J and 3M contain a deleted region on the p arm of chromosome 7 in **Patient One**. Similarly, Figures 3B, 3E, 3H, 3K and 3N display the data for a duplication on the p arm of chromosome 21 in **Patient One**. While Figures 3C, 3F, 3I, 3L and 3O show the presence of autozygous regions on chromosome 1 of a consanguineous individual. Regions of copy number variation can be seen as a series of Log_2_ values that do not tend to zero (Figures 3A, 3B and 3C). These values are then used to create the Smooth signal data (Figures 3D, 3E and 3F). Finally, the CN state for each probe is determined and shown as an integer value between 0 and 4 (Figures 3G, 3H and 3I). The genotype of each SNP probe is shown in the Allele difference dataset, which typically contains three clusters of values representing the ‘AA’, ‘AB’ and ‘BB’ genotypes. Deletions can be seen where data points form just two distinct clusters representing the A- and B- haploid genotypes (Figure 3J), while duplications are seen as four clusters of data points representing the AAA, AAB, ABB and BBB triploid genotypes (Figure 3K). Autozygous regions can be determined by the absence of the central heterozygous cluster (Figure 3L). LOH data points have a value of ‘Y’es or ‘N’o, with duplicated or deleted regions scoring N while autozygous regions or typically scored as Y.

To demonstrate the program's usefulness, we used CNViewer to identify a deletion distal to *PAX6* that co-segregates with individuals affected by aniridia in one family. We also visualised copy number data derived from two patients who presented with severe developmental problems caused by large scale *de novo* chromosomal re-arrangements. For comparison, these patients had previously undergone aCGH analysis, which is currently the method of choice for CNV detection in many clinical settings. Finally, we also demonstrate the detection of a 160 Kb deletion containing the *DPY19L2* gene in a patient who presented with suspected globozoospermia.

**Table 1:** Comparison of the regions of CNV in Patients One and Two, identified by oligonucleotide aCGH and regions identified by data derived from Affymetrix SNP 6.0 micro-array data.

Patient	Chromosome	Oligonucleotide aCGH			Affymetrix and CNViewer		
		Type	Interval (Mb)	Size (Mb)	Type	Interval (Mb)	Size (Mb)
**One**	7	Del	152.2 to158.8	6.6	Del	152.5 to 159.1	6.6
**One**	21	Dup	36.8 to 46.9	10.1	Dup	37.9 to 48.1	10.2
**Two**	15	Dup	83.7 to 102.5	18.8	Dup	84.0 to 102.5	18.5

## Results

### Data visualisation and analysis

Copy number and LOH information derived from the Affymetrix SNP 6.0 microarray contains both processed information (CN state; copy number, and LOH), raw data (Log2Ratio; copy number data and allele difference; loss of heterozygosity) and partially processed data (Smooth signal; copy number). CNViewer displays each of these data sets as a graph, containing information for a single chromosome, with the Y-axis indicating the probe's value and the X-axis identifying the probe's physical (in base pairs) position on the chromosome. If the genomic localisation of the genes and/or the cytogenetic bands on each chromosome are also loaded and included in the analysis, such information is shown below the data graphs. While LOH data points in the imported data originate solely from the SNP genotype probes, the copy number data is derived from both the copy number probes and the SNP genotype probes. Consequently it is possible to view the copy number data derived solely from the copy number probes, the SNP genotype probes, or both probe sets.

If the analysis involves input from multiple individual subjects, it is possible to highlight the data for each individual one at a time, by selecting the appropriate file name from the list contained in the ‘Overlay options’ panel. However, when viewing either the CN state or LOH data for multiple individuals, it may be more informative to select the ‘Show linked’ option. This option highlights the probes that have a common value in the affected patients but are not present in unaffected control patients.

Sub-microscopic structural variants are commonly found in individuals with no known genetic disease. However, these variants rarely exceed 100 Kb in length [3], [4]. Consequently, when large deletions/duplications (>1 Mb) are detected, it is not necessary to compare the patient's data with data derived from control individuals, since a large deletion or duplication containing multiple genes is unlikely to be benign. Therefore CNViewer allows such data from a single patient to be viewed.

For example, Figure 1A shows the ‘Smooth signal’ values across a ∼6.5 Mb deletion affecting approximately 18 genes on chromosome 7 of **Patient One**. However, as the size of the structural variant decreases, it becomes increasingly difficult to distinguish a pathological change from a non-pathological variant. Therefore, CNViewer can also display patient data with reference to data from unaffected individuals. This allows the selection of variants that are present in all the affected individuals, but are absent from unaffected control individuals. Thus, Figure 1B highlights the ‘Smooth signal’ data points for the affected and unaffected individuals in **Pedigree One** (upper and lower graphs, respectively) spanning the first 70 Mb of chromosome 11. It can be seen, that at a number of locations the data points diverge from the expected copy number value of 2. The structural features that are present in both affected and unaffected individuals, such as those highlighted by the red box in Figure 1B, can be discounted as being pathogenic, while those not present in the unaffected individuals cannot be discounted (highlighted by the blue boxes in Figure 1B). However, if the ‘CN state’ data for chromosome 11 is viewed with the ‘Show linked’ option selected, only a single region starting at 31.7 Mb is highlighted (red bar in Figure 1C). When this region is expanded, it can be seen to contain a deletion that is present in all the affected individuals, but absent from all the unaffected individuals (Figure 1D). To allow further analysis of a selected region it is possible to export the underlying data as a tab-delimited text file, which can easily be viewed in a spread sheet application such as Excel. A full description of the use of CNViewer is given in the user guide at http://dna.leeds.ac.uk/cnviewer/.

#### Pedigree One

When the Affymetrix SNP 6.0 copy number data for the five affected and three unaffected members of **Pedigree One** were analysed by CNViewer, only a single 0.57 Mb region of copy number variation was found to segregate with the disease phenotype (Figure 1B to 1D). Starting at 31.7 Mb of chromosome 11, this region was distal to *PAX6* and while it did not affect the transcribed regions of the *PAX6* gene, it did contain the *D11S2001* microsatellite, the *DCDC1*, *DNAJC24* and *IMMP1L* genes, and the 5′- coding sequences of the *ELP4* gene. Deletions distal to *PAX6* have previously been shown to cause aniridia [20], [21], [22] and are thought to inhibit the expression of the associated *PAX6* allele due to changes in the local chromatin structure.

### Data selection guidelines when using CNViewer

#### Dominant Inheritance

CNViewer is able to identify regions of copy number variation that segregate with a disease phenotype. Analysis of the inheritance pattern of aniridia in Pedigree One strongly suggested that the disease-causing mutation acted in a dominant manner and had complete penetrance. Consequently, it was possible to include unaffected siblings in the analysis. Since the exclusion power of an affected patient is the same as an unaffected sibling, in this case adding unaffected siblings significantly increased the exclusion power of the family. However, if the disease does not show complete penetrance, unaffected relatives should not be included for analysis, since they could lead to the exclusion of the disease locus.

#### Recessive Inheritance

Unlike dominantly-inherited diseases, the exclusion power of affected and unaffected individuals is not the same in recessively-inherited diseases. An affected individual must inherit a disease allele from both parents, whereas an unaffected child can inherit either no disease alleles or just one from either the mother or father. Consequently, there is a 1 in 4 chance that two alleles co-segregate with the disease phenotype, resulting in an individual affected by a recessive disease deing more informative than an individual affected by a dominant disease. Conversely, unaffected siblings of patients affected by a recessive disease are less informative than siblings of patients affected by a dominantly-inherited disease. Therefore, while the inclusion of unaffected siblings may help to reduce the number and size of candidate disease loci, in recessively-inherited conditions it is more important to include data from affected patients than their unaffected siblings. For a fuller description for the exclusion power of CNViewer when analysing recessive and dominant pedigrees is given in the supplementary document Text S1.

#### Patient One

Analysis of the copy number data for **Patient One** highlighted the two large structural variations previously identified by oligonucleotide aCGH. These variants consisted of the deletion of the telomeric region of the short arm of chromosome 7 (152.1 to 159.0 Mb, Figure 2A) and the duplication of the telomeric region of the long arm of chromosome 21 (36.8 to 47.0 Mb, Figure 2B).

#### Patient Two

Analysis of the copy number data for **Patient Two** identified a single duplication of the telomeric region of the long arm of chromosome 15 (81.8 to 100.3 Mb, Figure 2C), consistent with findings for oligonucleotide aCGH analysis.

#### Patient Three

Since the patient was consanguineous, the autozygosity status of the known disease loci was ascertained. Only the *DPY19L2* locus was found to lie in an autozygous region, strongly implicating this locus as pathogenic. Analysis of copy number status across the possible disease loci identified a 160 Kb homozygous deletion that spanned the *DPY19L2* gene (chromosome 12, 63,952,693 bp to 64,062,354 bp) (Figure 2D). Together, the autozygosity mapping and CNV data strongly suggest that this is the causative mutation in this individual.

### Identification of regions of autozygosity, hemizygosity and uniparental disomy

Figure 3 contains 3 series of images that display the graphs generated by CNViewer for deleted (Figures 3A, 3D, 3G, 3J and 3M), duplicated (Figure 3B, 3E, 3H, 3K, and 3N) and autozygous (Figure 3C, 3F, 3I, 3L and 3O) regions for each of the five different data value types. While regions containing a deletion (Figure 3G) are identified as having a copy number of 1 (CN state), they are not highlighted as regions of loss of heterozygosity (LOH) (Figure 3M). However, autozygous regions in consanguineous individuals are identified as having LOH (Figure 3O). Consequently, users who are interested in identifying regions of hemizygosity caused by allele loss should identify regions with CN state values of 1 and not use the LOH data set. However, the LOH data does identify regions of autozygosity and uniparental disomy, which are not detected by the CN state data points.

### Identification of copy number variants not associated with known disease loci

To demonstrate the ability of CNViewer to aid the detection of copy number variation not associated with a known disease locus, 5 sets of randomly selected data files where created such that each set contained two files assigned as ‘Affected’ and two files assigned as ‘Unaffected’. Each set was then manually screened for naturally occurring copy number variants (not using CNViewer) that were present in both the ‘Affected’ files but not in either of the ‘Unaffected’ files of at least one set. Each copy number variant spanned at least 6 consecutive probes and was not part of a larger copy number variant that did not co-segregate. These copy number variants were then used to create a group of 26 segregating copy number variants with lengths between 236 to 140,290 bp, and containing between 6 to 70 probes (Table S1). Two CNViewer users where then asked to identify all the copy number variants in the sets using only the CN state data values. Both users identified very similar sets of copy number variants each containing all the previously identified copy number variants. Regions identified by one user but not the other were subsequently found to be due to different selection criteria, with one user disregarding small regions of copy number variation if they were linked to larger CNV regions that did not segregate.

## Discussion

Copy number variation has generally been examined in the context of genome-wide association studies [23] and cancer genomics [24], [25], resulting in the development of software applications that are not suited to identifying regions of CNV that segregate with a disease phenotype in a pedigree or nuclear family. Consequently, we developed CNViewer to aid the visualisation of CNV data derived from Affymetrix's SNP 6.0 genotyping micro-array.

When used to screen CNV data from 5 affected and 3 unaffected members of a pedigree affected by aniridia, CNViewer identified a single region of CNV that co-segregated within the affected individuals. This deletion was found to be distal to *PAX6*, a region where similar deletions have previously been found in patients affected by aniridia [20], [21], [22].

When CNViewer was used to visualise CNV data from **Patients One** and **Two** with severe developmental problems, it was able to quickly identify the same regions as those found by oligonucleotide aCGH. When CNV data from **Patient Three** was observed using CNViewer across the known globozoospermia disease loci, only the DPY19L2 locus was found to be affected by a homozygous deletion. Since the other known disease loci appeared normal, this strongly suggests that this deletion is the cause of globozoospermia in this patient.

CNViewer can aid the rapid detection of large (>1Mb) regions of copy number variation and smaller regions linked to a known disease-causing locus. However, while it is also able to detect regions of copy number variation not linked to a known disease locus, when doing so it is important to decide on the minimum number of probes that will delimit a copy number variant and how regions connected to larger, none-segregating regions are treated.

As with aCGH, CNViewer analysis identifies regions of copy number variation, but does not identify the mechanism by which the variants cause a phenotype. For example, a duplication may give rise to a phenotype by affecting the expression of a gene present in the duplication or at the site of the duplication's insertion. Consequently, if a region of increased copy number is found to segregate with a disease phenotype, it will still be necessary to identify the exact mechanism by which the duplication causes or influences the phenotype.

While aCGH is the method of choice for copy number analysis for many clinical geneticists, the fact that current SNP genotyping microarrays offer the ability to both identify CNVs and genotype patients at no extra expense means that this technique is likely to become more important by identifying CNV linked to disease phenotypes. This can be seen in **Patient Three**, where a combination of both autozygosity mapping and identification of regions of copy number variation strongly implicated the deletion of a known gene as the cause of the patient's condition. While other programs, such as CNAG [14] and Affymetrix's Genotyping Console can visualise copy number data, CNViewer was developed to provide a more user-friendly system that can rapidly and easily identify CNV associated with a disease phenotype. CNViewer should become a useful addition to the toolbox of the clinical geneticist.

## Materials and Methods

### Ethical standards

Informed written consent was obtained from all adult participants and the parents or guardians of minors or children, and the study was approved by the Leeds (East) Research Ethics Committee (REC ref. no. 08/H1306/85).

### Software development and requirements

CNViewer has been tested on Microsoft Windows XP SP3, Vista SP1 and Windows 7, and requires the installation of the. NET framework 2.0. The program, user guide and sample files are freely available for download at http://dna.leeds.ac.uk/cnviewer/ and https://sourceforge.net/projects/cnviewer/.

### Data requirements

CNViewer is designed to analyse data derived from the copy number analysis of Affymetrix SNP 6.0 microarrays performed by the Affymetrix Genotype Console software. Although it is possible to export copy number, log2 ratio, smoothed signal, loss of heterozygosity (LOH) and allele difference values from the Genotyping Console, CNViewer does not require that all these fields be included in the exported data set.

### Patients

To demonstrate the ability of CNViewer to correctly identify regions of copy number variation segregating with a disease phenotype, we used the Affymetrix Genotyping Console to infer the copy number and LOH values from Affymetrix SNP 6.0 microarray data (Aros Applied Biotechnology A/S, Denmark) derived from the individuals described below.

**Pedigree One** (Figure S1) consists of two related nuclear families affected by aniridia (MIM# 106210), congenital absence of the iris associated with cataracts, corneal changes, and macular and optic nerve hypoplasia. This condition is known to be caused by dominantly-acting mutations in *PAX6* [26]. When the *PAX6* exonic sequences in the affected members of the pedigree were sequenced, no mutations were found. However, microsatellite analysis with the marker *D11S2001*, suggested that the affected patients were heterozygous for a deletion close to, but beyond the previously recognised 3′-extremity of *PAX6*.

**Patients One and Two** were referred for investigation of severe physical and mental developmental problems. These patients had previously undergone diagnostic oligonucleotide aCGH analysis using the Human Genome CGH Microarray Kit 44B (Agilent Technologies, Wilmington, DE) as described by Fan et al. [27], performed by an accredited service provider (Cytogenetic and Molecular Diagnostic Laboratory, Miller School of Medicine, University of Miami). This analysis had identified at least one large *de novo* chromosomal rearrangement in each patient (Table 1).

**Patient Three** was a consanguineous individual referred for investigation having received a preliminary diagnosis of globozoospermia (MIM# 613958). This condition had previously been linked to disruption of the human *SPATA16*, and mouse *Gopc* or *Pick1* genes [28], [29], [30] or the deletion of the *DPY19L2* locus mediated through the presence of low copy number repeats flanking that gene [31].

## Supporting Information

Figure S1 Figure S1A shows the structure of Pedigree One, which consists of two related nuclear families affected by aniridia. The asterisk by the patients' ID numbers identifies individuals for whom CNV data was collected. Microsatellite sizes for the marker D11S2001 are shown below each pedigree symbol. Figure S1B shows the structure of a hypothetical consanguineous pedigree in which 3 out 6 siblings are affected by a recessive condition.
(TIF)

Table S1 **From a collection of 12 copy number data files, 5 sets of files were created such that each set had two files assigned as affected and two files assigned as unaffected.** These sets where then manually screened (not using CNViewer) for naturally occurring copy number variant, in the autosomal chromosomes, which were present in both affected files, but not the unaffected files of a set. A set of 30 naturally-occurring copy number variants were then used to test the ability of three users to identify the previously identified copy number variants in each set.
(DOC)

Text S1 **Exclusion power of CNViewer when analysing dominant and recessive pedigrees.**
(DOC)
