# Supplementary figures and images for: Rapid Visualisation of Microarray Copy Number Data for the Detection of Structural Variations Linked to a Disease Phenotype
Source: PLoS One. 2012 Aug 17;7(8):e43466. doi: 10.1371/journal.pone.0043466 (PMC3422275; doi:10.1371/journal.pone.0043466)

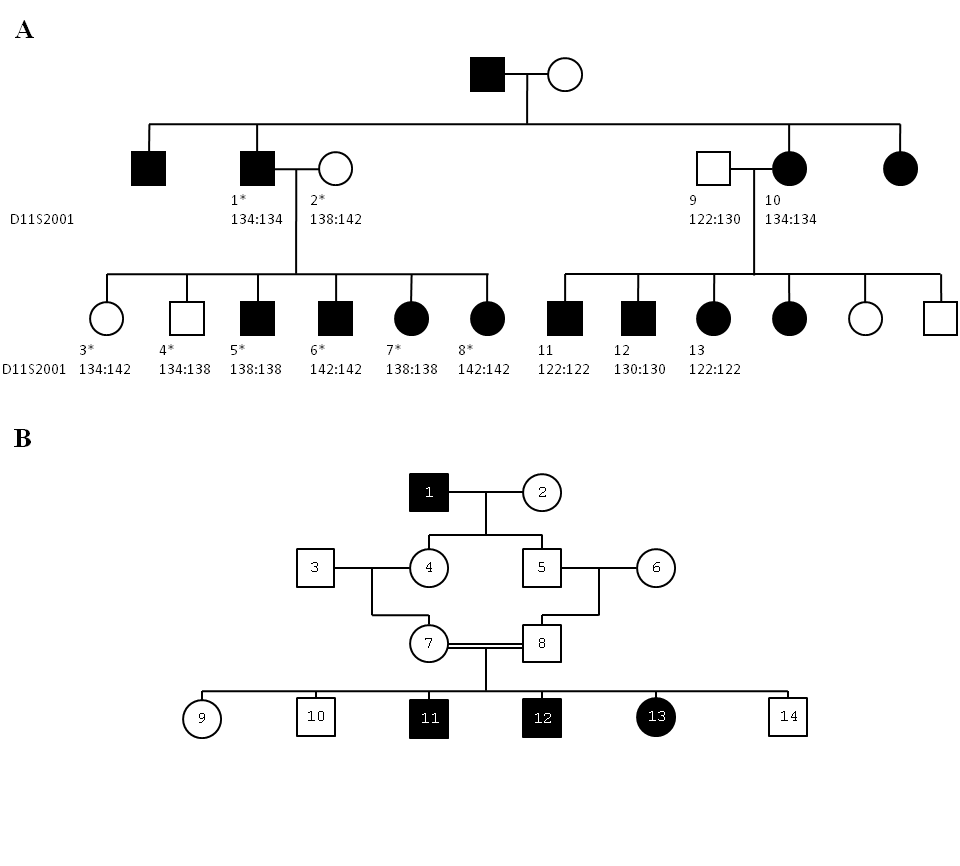

Supplement: Figure S1 — A shows the structure of Pedigree One, which consists of two related nuclear families affected by aniridia. The asterisk by the patients' ID numbers identifies individuals for whom CNV data was collected. Microsatellite sizes for the marker D11S2001 are shown below each pedigree symbol. Figure S1B shows the structure of a hypothetical consanguineous pedigree in which 3 out 6 siblings are affected by a recessive condition. (TIF) [file pone.0043466.s001.tif]
