# Supplementary material for: Rapid Visualisation of Microarray Copy Number Data for the Detection of Structural Variations Linked to a Disease Phenotype
Source: PLoS One. 2012 Aug 17;7(8):e43466. doi: 10.1371/journal.pone.0043466 (PMC3422275; doi:10.1371/journal.pone.0043466)
Supplement: Table S1 — From a collection of 12 copy number data files, 5 sets of files were created such that each set had two files assigned as affected and two files assigned as unaffected. These sets where then manually screened (not using CNViewer) for naturally occurring copy number variant, in the autosomal chromosomes, which were present in both affected files, but not the unaffected files of a set. A set of 30 naturally-occurring copy number variants were then used to test the ability of three users to identify the previously identified copy number variants in each set. (DOC) [file pone.0043466.s002.doc]

| Set | Affected  file IDs | Unaffected  File IDs | Chr | Region  Start (bp) | Region  End (bp) | Region  size (bp) | Number  of probes | Copy  number |
| --- | --- | --- | --- | --- | --- | --- | --- | --- |
| 1 | 09, 07 | 05, 08 | 3 | 80,466,481 | 80,470,231 | 3,750 | 6 | 4 |
| 1 | 09, 07 | 05, 08 | 3 | 162,512,645 | 162,625,983 | 113,338 | 70 | 4 |
| 1 | 09, 07 | 05, 08 | 7 | 53,466,013 | 53,590,735 | 124,722 | 71 | 1 |
| 1 | 09, 07 | 05, 08 | 7 | 133,785,183 | 133,790,431 | 5,248 | 25 | 3 |
| 1 | 09, 07 | 05, 08 | 12 | 30,237,497 | 30,243,710 | 6,213 | 26 | 3 |
| 1 | 09, 07 | 05, 08 | 12 | 116,237,579 | 116,246,822 | 9,243 | 28 | 3 |
| 1 | 09, 07 | 05, 08 | 18 | 63,200,345 | 63,204,705 | 4,360 | 15 | 4 |
| 1 | 09, 07 | 05, 08 | 22 | 22,382,096 | 22,382,332 | 236 | 6 | 4 |
| 2 | 05, 08 | 01, 06 | 3 | 162,512,645 | 162,625,983 | 113,338 | 69 | 3 |
| 2 | 05, 08 | 01, 06 | 20 | 1,561,568 | 1,593,837 | 32,269 | 43 | 4 |
| 3 | 01, 03 | 02, 04 | 5 | 46,271,918 | 46,273,490 | 1,572 | 6 | 0 |
| 3 | 01, 03 | 02, 04 | 6 | 32,456,671 | 32,518,757 | 62,086 | 17 | 4 |
| 3 | 01, 03 | 02, 04 | 12 | 90,491,657 | 90,492,062 | 405 | 7 | 1 |
| 4 | 10, 02 | 11, 12 | 1 | 10,410,9226 | 104,159,372 | 50,146 | 25 | 4 |
| 4 | 10, 02 | 11, 12 | 5 | 15,719,222 | 15,720,585 | 1,363 | 11 | 1 |
| 4 | 10, 02 | 11, 12 | 7 | 142,465,283 | 142,493,638 | 28,355 | 50 | 3 |
| 4 | 10, 02 | 11, 12 | 8 | 134,172,784 | 134,173,274 | 490 | 9 | 4 |
| 4 | 10, 02 | 11, 12 | 9 | 44,727,847 | 44,866,028 | 138,181 | 33 | 3 |
| 4 | 10, 02 | 11, 12 | 13 | 23,629,198 | 23,633,503 | 4,305 | 13 | 0 |
| 4 | 10, 02 | 11, 12 | 18 | 4,979,612 | 4,980,165 | 553 | 6 | 4 |
| 4 | 10, 02 | 11, 12 | 21 | 20,057,678 | 20,077,864 | 20,186 | 23 | 3 |
| 5 | 01, 02 | 11, 08 | 2 | 52,754,456 | 52,781,530 | 27,074 | 40 | 4 |
| 5 | 01, 02 | 11, 08 | 6 | 103,737,964 | 103,752,161 | 14,197 | 26 | 1 |
| 5 | 01, 02 | 11, 08 | 8 | 39,246,663 | 39,386,953 | 140,290 | 56 | 4 |
| 5 | 01, 02 | 11, 08 | 14 | 22,965,025 | 22,978,377 | 13,352 | 37 | 3 |
| 5 | 01, 02 | 11, 08 | 18 | 4,979,612 | 4,980,165 | 553 | 6 | 4 |

Table S1
