# Supplementary material for: Rapid Visualisation of Microarray Copy Number Data for the Detection of Structural Variations Linked to a Disease Phenotype
Source: PLoS One. 2012 Aug 17;7(8):e43466. doi: 10.1371/journal.pone.0043466 (PMC3422275; doi:10.1371/journal.pone.0043466)
Supplement: Text S1 — Exclusion power of CNViewer when analysing dominant and recessive pedigrees. (DOC) [file pone.0043466.s003.doc]

**Text S1**

**Exclusion power of CNViewer**

Dominant Inheritance: For dominantly-acting mutations, the chance that a particular inheritance pattern occurs by chance in a nuclear family is equal to 0.5^n^, where n is the number of siblings in the analysis. In the case of the analysis performed on siblings 3 to 8 in **Pedigree One**, the chance that this inheritance pattern occurred by chance is 0.5^6^ = 0.0156 which gives a LOD score of 1.8 [-(log_10_(0.5^6^))]. The chance that inheritance in the second nuclear family in Pedigree One (individuals 9 to 13) occurred by chance is 0.5^2^ = 0.125 (LOD score of 0.9). If it was not known that the two nuclear families were related, then the possibility that the inheritance pattern occurred by chance would be 0.0156 * 0.125 = 0.00195 (LOD score of 2.71). However, since the two nuclear families were related then the chance that the affected parents in each family inherited the same allele is 0.5. Therefore by knowing that these nuclear families were related, the probability that the segregation pattern shown by the deletion would be expected to occur by chance would be 0.0156 * 0.125 * 0.5 = 0.00098 (LOD score 3.01).

Recessive Inheritance: The chance that two deleterious alleles co-segregate with the disease phenotype in a nuclear family is 0.25^n^, while the chance that an unaffected sibling does not contain two disease alleles is 0.75^m^, where n is the number of affected siblings and m is the number of unaffected children. In the hypothetical nuclear family with three affected and three unaffected siblings (Figure S1B) the chance that two disease alleles co-segregate with the disease phenotype is (0.25^3^) * (0.75^3^) = 0.053 (LOD score 1.28). It is possible to increase the exclusion power of a nuclear family if more information is known about the occurrence of the disease in the parents’ ancestors. For instance, if the parents of the nuclear family described above are known to be first cousins (Figure S1B), the chance that both parents carry the same deleterious allele, inherited from a common grandparent is 1/16 (0.0625). Consequently, the chance that a locus is autozygous in the affected siblings, but not autozygous in the unaffected siblings is 0.0625 * 0.053 = 0.0033 (LOD score 2.47).
